# Combining parallel pattern generation of electrohydrodynamic lithography with serial addressing

**DOI:** 10.1039/c8ra06160b

**Published:** 2018-09-03

**Authors:** F. Boudoire, S. Partel, R. Toth, J. Heier

**Affiliations:** Empa, Swiss Federal Laboratories for Materials Science and Technology, Laboratory for Functional Polymers Überlandstrasse 129, 8600 Dübendorf Switzerland Jakob.Heier@empa.ch; Empa, Swiss Federal Laboratories for Materials Science and Technology, Laboratory for High Performance Ceramics Überlandstrasse 129, 8600 Dübendorf Switzerland; Vorarlberg University of Applied Sciences Vorarlberg Hochschulstrasse 1 6850 Dornbirn Austria

## Abstract

Electrohydrodynamic lithography (EHDL) is a parallel patterning process which typically makes use of topographically structured electrodes to guide pattern formation along areas of higher electrical field strength. The main driving force for pattern formation is an electrostatic pressure acting on a thin film polymer surface caused by a voltage applied between a top and bottom electrode. We here demonstrate that the principle can be applied using an addressable electrode composed of interdigitated fingers. Depending on the applied voltages, line patterns with different periodicities were fabricated. Our proof-of-concept experiments pave the way for a parallel pattern replication process where a serially addressed master is used. We complement the experiments by modelling the potentials across the electrodes and electrostatic forces acting on the polymer surface using different addressing schemes. Numerical simulations of the experimental setup pointed to some critical issues we experienced during the design of the experiments.

## Abstract 2

*Via* different electrode addressing schemes in electrohydrodynamic lithography, different patterns were generated.

## Graphical abstract

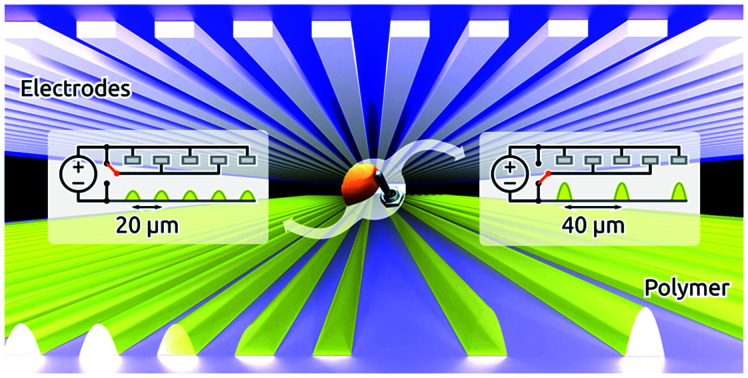

## Introduction

Cost-efficient production of miniaturized devices depends largely on the ability to pattern functional materials at desired length scales. In top-down approaches, one can differentiate between parallel and serial processes. Parallel methods such as photolithography or imprint lithography have the potential of high-throughput, which comes at the expense of flexibility since they require new masks or templates for any variation in the desired pattern. Differently, in serial lithography, such as e-beam lithography, the pattern is created by an e-beam that scans deterministically over the sample. Hence, any type of pattern can be defined, but this method is often unsuitable for mass production.

Yet another field of patterning strategies is based on bottom-up approaches. In this field the processes that compete with lithography frequently make use of instabilities of liquids or polymer melts and are referred to as field-guided assembly. A prominent technique is electrohydrodynamic lithography (EHDL) where an external electric field leads to self-assembly. In his pioneering work Steiner *et al.*^1,2^ has shown that a liquid film subjected to a uniform electric field destabilizes into periodic pillars whereby the spacing is determined by interfacial tension, applied voltage and film geometries. Long-range order in these films is weak and polymers destabilized between plane electrodes show at best hexagonal packing. Nevertheless, ordered patterns can be obtained with this method using a topographically patterned electrode. At the protruding structures of the electrode the electric field strength is higher, leading to a higher growth rate of the instability. In the best case, the electrode pattern is positively replicated in the polymer film.^3–7^ Efforts to decrease feature size range from increasing applied voltage and decreasing gap distance^2^ to lowering the interfacial tension.^6^ Recent papers elaborate on improvements in aspect ratio^8,9^ and summarize the parameters influencing the faithfulness of pattern replication.^10^

A strong aspect of EHDL is that a wide range of materials can be directly patterned. Patterns of titanium oxide have been created by combining sol–gel processing with EHDL.^11,12^ The principles of EHDL have also been applied to molten films of metals and semiconductors.^13^ EHDL has been applied in the fabrication of “nano-hair”,^14^ micro-electro-mechanical systems (MEMS) such as micro-fluidic mixers^15^ and arrays of micro-lenses.^16^

Following the existing literature, electrical field strength variation has been almost exclusively realized by introducing topographical features to the electrode. In one report patterning flexibility was introduced by irradiating a photosensitive layer with light defining the structure.^17^ However, electrical field strength being the main driver for film destabilization, EHDL offers the unique opportunity to combine a parallel patterning process with a flexibly patternable template. With a single addressable master electrode a large number of different patterns could be generated. Moreover, for template fabrication one can rely on the highly advanced technology of electrode arrays utilized in displays^18^ and biotechnology applications.

In the following we demonstrate the working principle of the idea with a simple proof-of-concept device. The structured electrode consists of two interdigitated electrodes that can be addressed individually. The resulting potential differences and electrostatic pressures acting across the device and at the polymer surface, respectively, are simulated. The model allowed us to overcome a caveat that we faced experimentally, leading to a failure of the pattern replication.

## Results and discussion

**Fig. 1:**
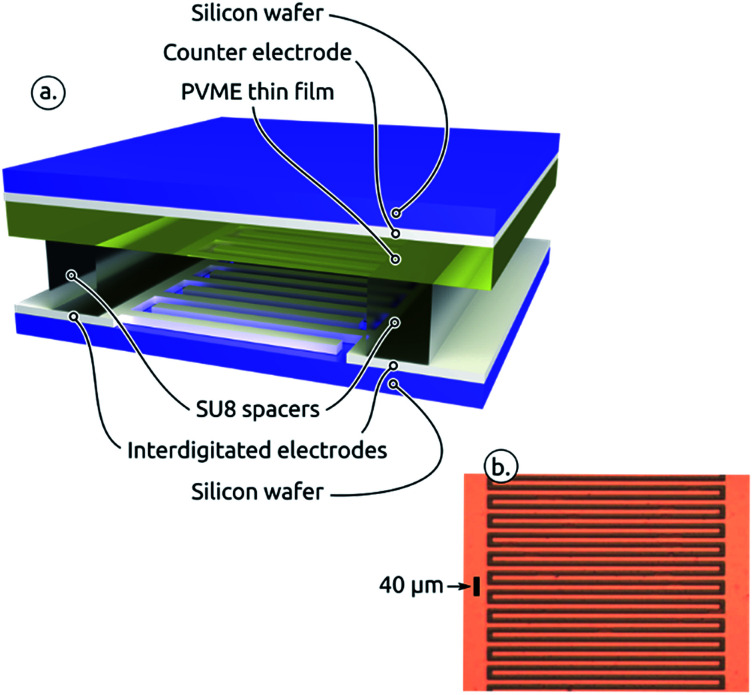
(a) Sketch of the experimental set-up. (b) Optical micrograph of the interdigitated electrodes. The conductor pattern appears orange.

The experimental setup used for pattern replication is shown in Fig. 1(a) and described in detail in the Experimental section. In the experiment a poly(vinyl methyl ether) (PVME) thin film is sandwiched between a silicon substrate supporting the interdigitated electrodes and a silicon substrate supporting a flat counter electrode. An air gap is created using SU8 spacers between the polymer film and the interdigitated electrodes. Fig. 1(b) shows a microscopic image (top-view) of the interdigitated structured electrode.

**Fig. 2:**
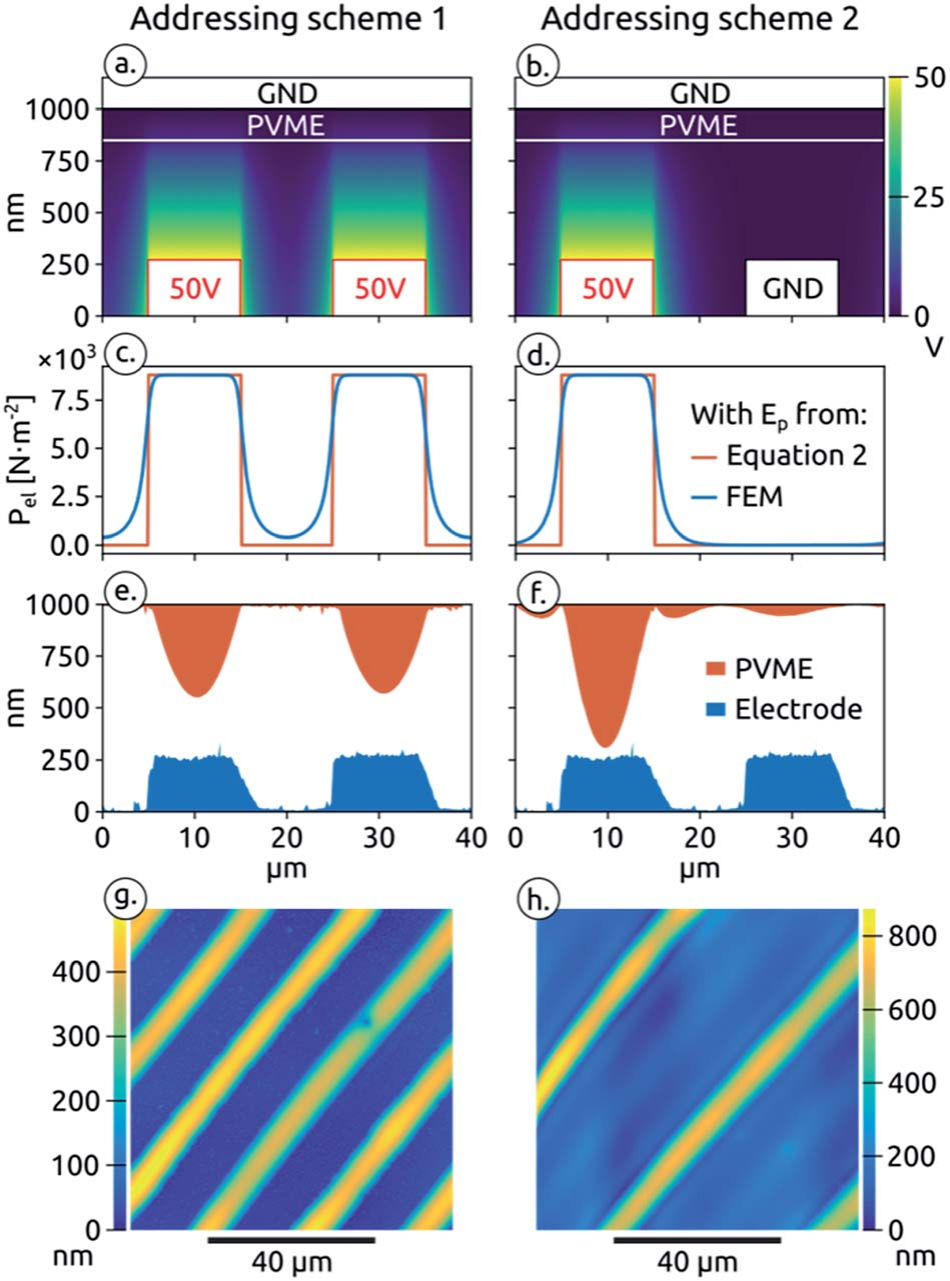
(a) and (b) Representation of addressing scheme 1 and 2 and potential distribution simulated by FEM; (c) and (d) electrostatic pressures derived from the electric field simulated by FEM and calculated using eqn (2); (e) and (f) AFM profiles of the destabilized PVME thin film and the interdigitated electrodes; (g) and (h) AFM images of the destabilized PVME film.

The interdigitated electrodes can be addressed individually, with two different addressing schemes, represented in Fig. 2(a) and (b) together with the resulting electrical potential distribution that was modelled using the finite element method (FEM). In the first addressing scheme (addressing scheme 1, Fig. 2(a)), a potential difference of 50 V is applied between the counter electrode and both interdigitated electrodes. In the second addressing scheme (addressing scheme 2, Fig. 2(b)), this potential difference is only applied between one interdigitated electrode and the counter electrode while the second interdigitated electrode is short-circuited to the counter electrode. The expectation is that depending on the applied addressing scheme a grating with a periodicity of 20 μm or 40 μm, respectively, is obtained.

The flow of a dielectric liquid in a thin film exposed to an electric field can be explained by the polarization of the film leading to a displacement of charges at the liquid–air interface. This displacement generates an electrostatic pressure *P*_el_ given by:^2^

1 *P*_el_ = −*ε*_0_*ε*_p_(*ε*_p_ − 1)*E*_p_^2^

whereby *ε*_0_ and *ε*_p_ are the dielectric constant of vacuum and polymer, respectively. The relative dielectric constant of PVME was set to 2.15.^19^ If a homogeneous electric field is applied to the polymer, local fluctuations in film thickness result in an electrostatic pressure gradient that drives lateral flow of the liquid. This pressure is counterbalanced by the Laplace pressure. A linear stability analysis accounting for the competition between these two pressure fields shows that of all film thickness perturbation wavelengths, one wavelength *λ*_m_ is amplified the fastest.^1,2^ Pattern control as described in the present study is achieved by controlling the electrostatic pressure distribution using our micro-structured addressable electrodes.

For the two addressing schemes we calculated *E*_p_ and subsequently *P*_el_ from the FEM simulation of the electrical potential distribution. The variations of *P*_el_ along the polymer surface calculated from the simulation results are presented in Fig. 2(c) and (d) (blue trace). Similar values of *P*_el_ (Fig. 2(c) and (d)), (red trace) are also obtained by considering our assembly at a given position as a capacitor under a constant potential and deriving *E*_p_ from the stored free energy,^6,11^ which lead to the following equation:2
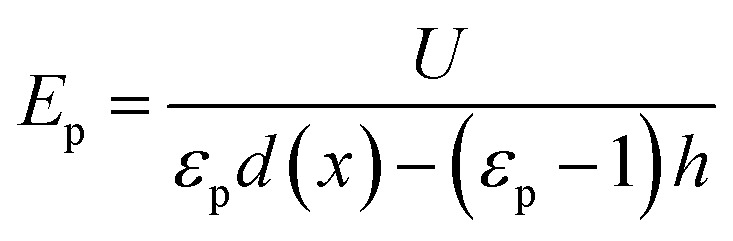
with *U* the applied voltage, *d* the distance between the counter electrode and the interdigitated electrodes and *h* the polymer film thickness.

With the first addressing scheme (Fig. 2(c)) a large electrostatic pressure is achieved in front of both interdigitated electrodes and should lead to a PVME grating of 20 μm periodicity. The simulation result shows that the potential obtained between the finger electrodes never drops to zero, creating an electrostatic pressure across the entire film. Still, the electrostatic pressure acting at the film surface is significantly lower between the electrodes (400 N m^−2^*vs.* 8800 N m^−2^) and does not jeopardize the pattern replication of both interdigitated electrodes. With the second addressing scheme (Fig. 2(d)) the electrostatic pressure becomes null in front of one of the interdigitated electrode. Therefore the polymer should follow the morphology of only one interdigitated electrode leading to a double grating periodicity of 40 μm.

The PVME patterns resulting from these different addressing schemes are presented in Fig. 2(e)–(h). The AFM scans show that the polymer microstructure followed the electrostatic pressure profile presented in Fig. 2(c) and (d) successfully. Our simple “pattern generation device” is therefore able to produce PVME gratings with different periodicities by just switching the potential of one addressable electrode from ground to +50 V. It is interesting to note that for a given PVME film thickness the limited volume of polymer available will lead to different pattern height. Indeed, the maximum height achieved for the 20 μm periodicity is 450 nm *versus* 690 nm for the 40 μm periodicity.

**Fig. 3:**
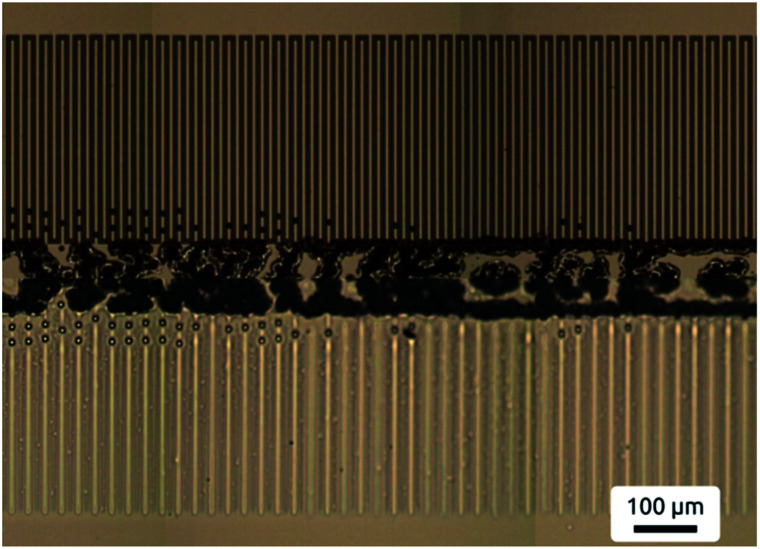
Microscopy image demonstrating the ease of pattern replication over larger areas. The bottom panel shows the PVME film, the top panel the electrode pattern. After pattern formation the two plates have been separated and the top electrode has been folded upwards. The features dividing top and bottom panel are the electrode spacers. The mirror plane character of the horizontal line is visible.

Moreover the destabilisation proved to occur with great fidelity over a larger scale as shown in Fig. 3, which is important when considering the use of this method for high-throughput pattern fabrication. The micrograph shows both, electrode pattern and polymer film after unfolding the electrode – film sandwich. (The top panel presents the two interdigitated electrodes with a total periodicity of 20 μm. The bottom panel shows the PVME film, reproducing every second line of the interdigitated electrodes with a periodicity of 40 μm, following the second addressing scheme).

**Fig. 4:**
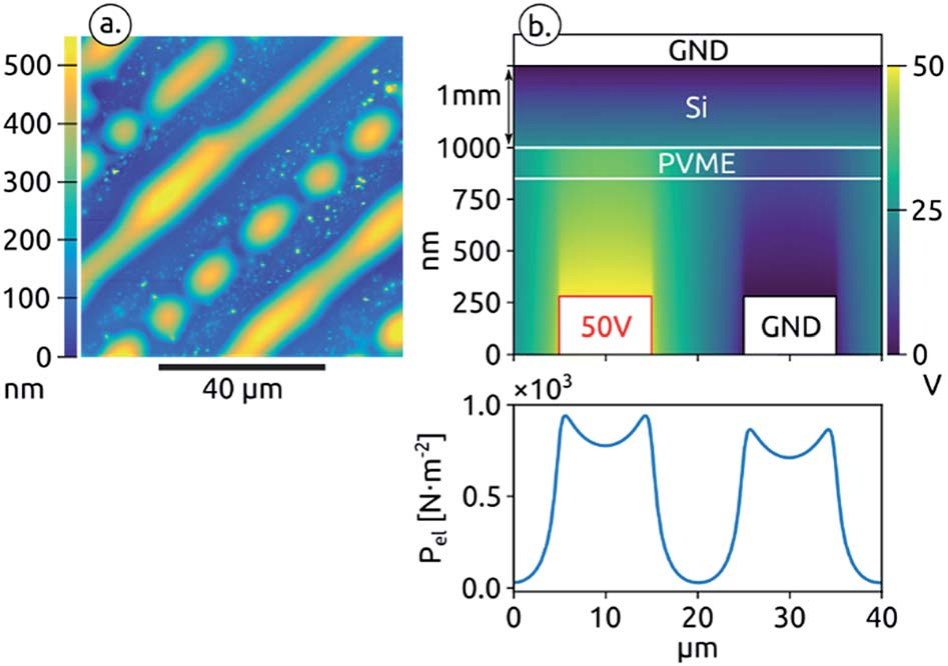
(a) AFM image of a PVME film destabilized with an assembly where the counter electrode is contacted on the opposite side of the silicon substrate (b) scheme of the assembly, electric potential distribution simulated by FEM and calculated electrostatic pressure at the polymer surface.

During the design of the assembly presented in Fig. 1(a) we discovered a crucial issue that can prevent the formation of the pattern with the larger periodicity (40 μm, addressing scheme 2), *i.e.* also forms a pattern above the electrode that is not addressed. In previous experiments we had contacted the back of the silicon wafers supporting the PVME films with silver paste, according to the scheme shown in Fig. 4(b), instead of depositing a flat conductive counter electrode below the polymer film, following the procedure which is usually adopted for EHD and EHDL experiments.^7,11^ Using this configuration we always obtained a 20 μm periodicity (Fig. 4(a)) instead of the 40 μm periodicity expected for this addressing scheme. The FEM simulation of the potential distribution gave precious insight in the cause of this phenomenon. For this simulation, presented in Fig. 4(b), we added a 1 mm thick dielectric slab, with a relative permittivity of 11.7, to represent the silicon substrate between the polymer and the counter electrode. The result of this simulation shows that the presence of a dielectric material between the counter electrode and the PVME thin film prevents the formation of a constant potential in front of the short-circuited interdigitated electrode. Instead, a potential gradient is established leading to the formation of an electric field and an electrostatic pressure builds in front of this electrode.

The electrostatic pressure distribution is shown in the bottom panel of Fig. 4(b) In this case the pressure is almost one order of magnitude lower compared to the case where the counter electrode is directly at the back of the PVME thin film. Still this pressure is enough to destabilize the PVME film in front of both electrodes as we observe experimentally (Fig. 4(a)). It is interesting to note that there is a slight dip in the pressure intensity in front of the interdigitated electrodes with a difference in mean pressure of around 70 N m^−2^. This result not only demonstrates the importance of a correct addressing of the electrodes, but also points to the challenges of exact pattern replication. Many of these challenges have been addressed theoretically.^4^ A general issue is the competition between the establishment of features with a wavelength *λ*_m_ set by the fluid properties (*e.g.* thickness, viscosity) and the applied electric field periodicity. Larger variations in electrostatic pressure will be more likely to overcome the flow caused by local film thickness variations, improving the pattern reproduction. In particular, in order to generate a certain feature one has to make sure that enough liquid is available to form it. In our case, the fragmentation of every second line into droplets is due to depletion of liquid towards the more stable ridges, in the region of higher field strength (Fig. 4(b)).

Interestingly, the detrimental effect of contacting the back of the silicon substrate instead of the face supporting the polymer thin film can only be observed in the case of our setup involving addressable electrodes. When only one master electrode is used to drive the destabilization, the pattern would be reproduced independently of which face was contacted.

## Experimental

### Structured electrodes

The electrodes were fabricated by standard photolithography and semiconductor techniques. A 100 mm silicon wafer was thermally oxidized (PEO 604, ATV Technologie GmbH, Germany). Afterwards, the 200 nm SiO_2_ layer is covered with a 200 nm aluminium layer by sputter deposition (LLS EVO, Oerlikon Advanced Technologies, Liechtenstein). A 2.4 μm thick layer of AZ 1518 photoresist (purchased from Microchemicals GmbH, Germany) was spin-coated and a softbake at 100 °C for 80 seconds was performed. The subsequent UV exposure was carried out in a Mask-Aligner (MA/BA6, SUSS MicroTec AG, Germany). A broadband illumination with a dose of 30 mJ cm^−2^ in vacuum contact mode was applied. The wafer was immersed in AZ726 MIF developer (purchased from Microchemicals GmbH, Germany) for 45 seconds and rinsed with deionised water, followed by a N_2_ blow dry. In a further step the aluminium layer was etched in aluminium Etchant (purchased from Microchemicals GmbH, Germany). The removal of the photoresist mask was performed with acetone followed by a rinse with isopropanol.

### Experimental set-up

The experimental set-up is shown in Fig. 1. A PVME film/air-gap is sandwiched between two electrodes, with the air-gap being located between structured bottom-electrode and polymer surface. A 1% wt solution of PVME in toluene (Scientific Polymer Products, USA) was spin coated at 3000 rpm directly on top of the Ag top-electrode resulting in a 150 nm thick PVME film. The counter electrode is a 50 nm thick film of Ag thermally evaporated on top of a supporting silicon wafer. PVME has a glass transition temperature of *T*_g_ = −20 °C. The generated topographical features have a limited stability due to relaxation of PVME. Nevertheless, the features remained stable for a couple of hours. The choice for this polymer was motivated by the fact that no annealing was necessary to liquefy the polymer film. To fabricate a well-defined air-gap between PVME and top-electrode a spacer was introduced on top of the structured electrode: parts of the electrode were covered with a Kapton film. The substrate was then coated with a SU8 (micro resist technology, Germany) solution (33% vol SU8 in SU8 thinner, spincoated at 3000 rpm and cured at 150 °C for 15 min). Spacers of 100 nm thickness were obtained after removal of the Kapton film. The two substrates were slightly pressed against each other using a custom setup and a voltage of 50 V was applied according to the specifications in the Results section.

### Characterization

Films were characterized by optical microscopy (Leica) and scanning force microscopy (Digital Instrument Nanoscope III MultiMode Scanning Probe Microscope).

### Simulations

The electrostatic modelling using the finite element method was performed using the Gmsh^20^ and GetDP^21^ software.

## Conclusions

We demonstrated the potential of EHDL to serve as a generic platform for direct surface structuring of topographical patterns combining the advantages of serial and parallel structuring processes. We performed proof-of-concept experiments with a very simple electrode addressing scheme composed of two interdigitated electrodes. From a practical side we showed that it was crucial that the polymer thin film is coated directly onto the counter electrode for this assembly to work as intended.

In the “ultimate pattern generator”, the electrode consists of an array of individually addressable pixels, in the same fashion as active or passive matrix schemes known from the display industry.

As EHDL relies on flow of material towards regions of high electrical field strength, in most reported applications all replicated features have similar characteristic dimensions (lateral size and height). For a more generic scheme, restrictions may exist how close the individual feature dimensions have to be. For a topographically structured template electrode it has been shown experimentally and theoretically that features varying in size over an order of magnitude can be formed simultaneously.^22^ Similarly, even hierarchical structures can be realized by EHDL.^23^

It should be also noted that the structuration method proposed in this study can be extended to almost any dielectric liquid. Critical materials parameters are for example the dielectric constant and surface tension, which determine the electric field strength necessary to destabilize the interface. Another critical parameter is the viscosity of the liquid that will set the timescale for the evolution of the pattern formation. For a polymer film, destabilization should occur well above their glass transition temperature (*T*_g_) in order to achieve a viscosity allowing for pattern formation at a relevant timescale. Hence, most polymer films need to be heated during destabilization. This renders the pattern replication more challenging to set up but allows to maintain the patterns upon cooling down below *T*_g_. The polymer PVME that we used for our studies is peculiar in this regards because it has a glass transition temperature below ambient temperature. Consequentially, in time the sample topography faded away. It would be interesting to study the reversibility of the patterning process and use it for the live adjustment of the polymer microstructure by changing the addressing scheme over time.

## Conflicts of interest

There are no conflicts of interest to declare.

## Supplementary Material
